# Effects of Suboptimally Presented Erotic Pictures on Moral Judgments: A Cross-Cultural Comparison

**DOI:** 10.1371/journal.pone.0158690

**Published:** 2016-07-01

**Authors:** Antonio Olivera-La Rosa, Guido Corradi, Javier Villacampa, Manuel Martí-Vilar, Olber Eduardo Arango, Jaume Rosselló

**Affiliations:** 1 Department of Psychology and Social Sciences, Fundación Universitaria Luis Amigó, Medellín, Colombia; 2 Human Evolution and Cognition Group, associated group to IFISC (University of the Balearic Islands–CSIC), Palma de Mallorca, Spain; 3 Department of Basic Psychology, University of Valencia, Valencia, Spain; University of Würzburg, GERMANY

## Abstract

Previous research has identified a set of core factors that influence moral judgments. The present study addresses the interplay between moral judgments and four factors: (a) incidental affects, (b) sociocultural context, (c) type of dilemma, and (d) participant’s sex. We asked participants in two different countries (Colombia and Spain) to judge the acceptability of actions in response to personal and impersonal moral dilemmas. Before each dilemma an affective prime (erotic, pleasant or neutral pictures) was presented suboptimally. Our results show that: a) relative to neutral priming, erotic primes increase the acceptance of harm for a greater good (i.e., more utilitarian judgments), b) relative to Colombians, Spanish participants rated causing harm as less acceptable, c) relative to impersonal dilemmas, personal dilemmas reduced the acceptance of harm, and d) relative to men, women were less likely to consider harm acceptable. Our results are congruent with findings showing that sex is a crucial factor in moral cognition, and they extend previous research by showing the interaction between culture and incidental factors in the making of moral judgments.

## Introduction

Moral judgments have become a major research topic in social cognition. The emerging science of moral psychology has shown that most moral judgments are the result of automatic processes [1– 3]. For instance, it has been argued that moral judgments are typically driven by affect-laden intuitions: in the presence of a moral event, we experience an instant feeling of approval or disapproval [1]. In the last fifteen years, several studies have focused on the susceptibility of moral judgments to individual and contextual factors, such as gender [4,5], sociocultural context [6, 7], type of dilemma [8] and incidental affective responses [9, 10].

First, research on the automaticity of social cognition has found new possibilities through the study of how incidental affects influence moral judgments. Moreover, according to Landy and Goodwin [11], the influence of affective factors on moral judgments is best tested when the affective induction is unrelated to the moral judgment in question. In fact, inducing feelings of disgust, through a hypnosis manipulation [10], a disgusting smell [3] or a bitter taste [9], increases the perceived wrongness of moral violations without participants’ awareness of the experimental manipulation. Recently, unpublished research from our laboratory showed that affective priming by highly arousing unpleasant pictures (depicting human mutilations) reduced the severity of moral judgments in a Spanish sample of participants, but did not influence the moral judgments of a Colombian sample, representing a population that is more habituated to violent stimuli. The apparent divergence between the particular effect of affective priming found in this research and previous studies seems to be a matter of methodological differences between experimental paradigms (see also [12]).

Second, with regard to the role of sociocultural differences in moral judgments, several studies from the field of anthropology and cultural psychology have demonstrated that morality cannot be properly understood without taking into account sociocultural factors. In this context, cross-cultural research on moral universals has shown that although some moral issues are virtually universal (e.g.; “it is wrong to produce harm without any kind of justification”), morality varies across cultures in many ways, such as moral concerns, norms, practices or values [13]. For instance, several cultures consider sexual regulations as an important part of the protection of the purity of the moral self [14]. Even in modern Western culture, sexual but harmless actions were judged differently depending on socioeconomic status or political affiliation [7, 15]. Moreover, it has been shown that moral judgments are influenced by social class, with upper-class participants being more likely to choose the utilitarian choice in moral dilemmas [6], a pattern of response that is associated with lower levels of empathy for others’ suffering [16].

Third, a growing body of studies from the field of neuroscience suggests that distinct contributions of affective and cognitive processes occur in the making of moral judgments. According to the dual-process model of moral judgments [2], the role of emotion and cognition in moral judgment varies depending on specific factors in the dilemma formulation. With regard to this issue, dilemmas in which the agent carries out the action by himself are considered “personal” moral dilemmas. Conversely, moral dilemmas in which the harm is not directly carried out by the agent, are classified as “impersonal” [2, 8]. Moreover, it is suggested that personal dilemmas favor deontological positions (which means that the wrongness of an action is context-independent) and impersonal dilemmas pit utilitarian reasoning (the wrongness of the action is judged in the light of its overall consequences). Even though the explanatory validity of the personal-impersonal distinction has been questioned [17], several studies have found support for this proposal [18–20].

Fourth, the role of sex differences in moral judgments is a central theme in moral psychological research. For decades, the dominant approach to this topic identified men with a rational pattern of moral decision and women with an emotional one [21]. Moreover, it has been stated that women’s moral judgments are more sensitive to concerns about care and moral purity, whereas men are more sensitive to issues related to fairness [5]. Although the current state of the art is mixed [22], recent studies found that women exhibited a stronger sense of moral identity and stronger deontological inclinations than men, which suggest that sex differences in moral judgments are mediated by differences in affective responses to harm [4, 23].

In light of the above findings, the present research attempts to go further by testing the effects of suboptimally presented affective priming using erotic pictures on moral judgments. Erotic stimuli are one of a kind among positive stimuli, in the sense that they are rated as both affectively pleasant and highly arousing by both men and women [24], and have proved to be one of the most attention-grabbing classes of stimuli [25], as well as being sensitive to factors such as context and gender [26; 27]. It has been suggested that, when the exposure to erotic stimuli is subliminal rather than supraliminal, it might increase the mental accessibility of sex-related information [28, 29]. On the other hand, previous findings suggest that supraliminal exposure to erotic stimuli involves further cognitive processing of such stimuli (e.g., elaborate appraisal processes) leading to unclear or conflicted responses [29]. Indeed, there is evidence suggesting that subliminal erotic stimuli reduce participants’ tendencies to activate regulatory processes, causing stronger effects on cognition than when exposure is above the threshold of awareness [30].

Interestingly, erotic stimuli may activate the experiential system, prompting participants to perceive freedom and responsibility as negatively correlated [31]. This activation seems, however, to be limited to men [32]. Moreover, there is evidence suggesting that sexual arousal can narrow the focus of motivation, creating a sort of “the ends justify the means” pattern of decision-making [33].

Therefore, it is intriguing to extend the study of the effects of erotic stimuli to the moral domain. With this aim, the present study addresses the interaction between four types of factors that are especially relevant in the making of moral judgments: sex, sociocultural context, type of dilemma and incidental affects. Specifically, given the fact that these four types of factors are known to influence moral judgments, we expect to find a main effect of each of them on the acceptance of harmful actions. Moreover, given the cross-cultural nature of the present research, an important issue concerns whether cultural differences will have an effect on the likelihood to judge harmful actions as acceptable. Following previous research on culture and morality [34, 14] we expect to find differences in moral judgments between two different countries. Additionally, in line with previous unpublished research showing that the effects of affective priming on moral judgments are modulated by cultural factors, we hypothesized that the effects of suboptimally presented erotic primes on the likelihood to accept harm for a greater good (i.e., utilitarian moral judgment) would be modulated both by characteristics of the sample (sex, culture) and the target (type of dilemma). First, following research on sex differences in the processing of visual erotic stimuli [26, 28], we expected that men would be more sensitive to erotic primes than women. Second, in line with previous unpublished research from our laboratory, we expected that Colombians would be less sensitive to the affective nature of the primes than Spaniards. Third, we expected that personal dilemmas (which are known to recruit more affective circuits in the brain) would be more sensitive to affective primes than impersonal dilemmas.

## Methods

### Participants

All participants were university students (*N* = 224) who were invited via internal mail to join the experiment as a part of their course credits. All participants gave written informed consent. The study was approved by the Bioethics Committee of the University of the Balearic Island (Spain), University of Valencia (Spain) and FUNLAM (Colombia). All participants had normal or corrected-to-normal vision and were between 18 and 22 years old (112 males, age *M* = 21.32 years, *SD* = 1.85). In order to perform the cross-cultural comparison we selected samples from two different countries: Spain and Colombia (*n* = 112 and *n* = 112, respectively).

### Materials and stimuli

We displayed the stimuli on a 20-inch screen (60Hz refresh rate) PC running OpenSesame v. 2.9.1 [35] on Microsoft Windows 8. We used fourteen erotic (pleasant-arousing) pictures from IAPS [36] (adapted to Spanish populations [37, 38] and to Colombian populations [39]) as erotic primes. In order to control for differences in participants’ sexual preferences relative to the content of the primes, we only selected those pictures in which both men and women were involved in the sexual act. Still, it is worth noting that dimensional differences between sexes remained in the ratings of the IAPS pictures in the dimensions of both valence (*p* < .001) and arousal (*p* < .001). As pleasant primes, we used 14 pictures selected from the IAPS (1024 x 768 pixels) following the criterion that they presented higher values in valence and middle values in arousal. We selected as neutral primes fourteen pictures from IAPS, following the criterion that they presented middle values in both valence and arousal (data in S1 Text). As targets, we selected 42 moral dilemmas, made up of 21 moral personal dilemmas and 21 moral impersonal dilemmas (from [40]; dilemmas in S2 Text). All vignettes were accompanied by a 7-point Likert scale ranging from 1 (completely wrong) to 7 (perfectly OK).

### Procedure

Participants rated a set of 42 dilemmas in a 2 (Sex: men *vs*. women) x 2 (Country: Colombia *vs*. Spain) x 3 (Type of Prime: neutral *vs*. pleasant *vs*. erotic) x 2 (Type of Dilemma: impersonal *vs*. personal) mixed design, with the participant’s sex and country as between-subject factors, with both type of prime and type of dilemma as within-subject factors, and with moral judgments as the dependent variable. Before each session, we asked all participants to sign a written consent form. Later, we proceeded with the experimental instructions. We emphasized that we were asking participants for their first reactions and that it was important to respond quickly.

The experimental paradigm consisted of 46 trials. Before the battery of dilemmas, we introduced four vignettes with instructions, followed by another four vignettes with dilemmas (two of them “personal” and two of them “impersonal”), in order to familiarize the participants with the dynamic of the experiment. We did not consider the ratings of these four dilemmas in the subsequent analyses. The experimental paradigm was a self-paced task, designed so that the next dilemma was not presented until the subject had responded to the previous one. The pairing of specific dilemma to prime type was randomized. Each trial started with the presentation of a fixation cross in the center of the screen for 500ms. After a short delay (ISI = 100ms), the targets (both personal and impersonal dilemmas) were presented in the form of written vignettes. We instructed participants to press the key-press response (space bar) on the keyboard once they finished reading each dilemma. Then, we presented the prime for 16ms, immediately followed by a noise-pattern backward mask (250 ms). The pattern-mask size was 1920 x 1080 pixels. A 7-point Likert scale ranging from 1 (completely wrong) to 7 (perfectly OK) was presented immediately at offset of the backward mask. Thus, higher ratings corresponded to more acceptance of causing harm for the greater good (more utilitarian judgments) in the evaluations of the vignettes. Although the presentation times for the masked primes were shorter than those used in prior studies reporting that participants were unable to detect subliminally presented erotic primes even after repeated presentations [28, 41], we asked participants to answer a self-report question (“Have you seen any picture appearing on the screen?”) after they completed the task. No-one reported having seen anything.

## Results

We analyzed data using both R statistical package [42] and SPSS 20.0.0 (SPSS Inc., Chicago, IL, USA). We set the alpha level at .05, except when conducting pairwise comparisons, for which Bonferroni adjustments were used. Eta-squared was used in order to compare differences in effect size.

Given the fact that both extremely short and extremely delayed response times can seriously affect the statistical analysis and further interpretation of the data, we first proceeded to examine the responses on a trial-by-trial basis, with reference to the corresponding response times. More specifically, because responses had to be based on participants’ initial impression, all observations with response times greater than the mean plus two SD were excluded from the final analyses (4.32% of all responses). Moreover, in order to avoid anticipated responses, we disregarded those trials with a response time lower than 300ms (2.12% of all responses). Finally, we restructured the remaining data (93.55% of responses) in wide format, setting the mean of Likert scores for each combination of the two intra-subjects factors (Type of Prime and Type of Dilemma) as the dependent variable. From this point on, we based analyses on the depurated data.

We checked the assumptions of normality and homogeneity of variances through the Shapiro-Wilks and Levene tests, respectively. Mauchly's test of sphericity was also conducted. Every assumption was properly met. We therefore conducted a mixed between-and-within-subjects 2x2x3x2 ANOVA to assess the effects of the between-subjects factors (Country: Colombia *vs*. Spain; Sex: men *vs*. women) on participants’ mean scores across the within-subjects factors (Type of Prime: neutral *vs*. pleasant *vs*. erotic; Type of Dilemma: impersonal *vs*. personal).

We found a main effect of Sex, *F*(1,220) = 11.163, *p* = .001, *η*^2^ = 0.051, 95% CI [0.008, 0.113]. The comparison between men and women showed a statistically significant mean difference (*MD*) of 0.518 (95% CI [0.212, 0.824]), with men (*M = 4*.*42*, *SD* = 1.18) showing higher Likert scores (i.e., evidencing more acceptance of harm/utilitarian moral judgments) than women (*M* = 3.902, *SD* = 1.116).

There was also a main effect of Country, *F*(1, 220) = 5.909, *p* = .016, *η*^2^ = 0.027, 95% CI [0.001, 0.080], indicating that the mean score for Colombian people (*M* = 4.35, *SD* = 1.184) was higher (i.e., more acceptance of harm/utilitarian moral judgments) than for Spanish people (*M* = 3.97, *SD* = 1.188), with a statistically significant *MD* of 0.377, 95% CI [0.071, 0.683].

Likewise, Type of Dilemma showed a statistically significant main effect, *F*(1,220) = 68.764, *p* < .001, *η*^2^ = 0.238 95% CI [0.147, 0.327], suggesting that participants were less likely to accept harm (the utilitarian judgment) when judging personal dilemmas (*M* = 4.04, *SD* = 1.244) than impersonal dilemmas (*M* = 4.281, *SD* = 1.194). More specifically, the statistically significant *MD* was 0.241, 95% CI [0.183, 0.3]

We also found a main effect of Type of Prime on moral judgments, *F*(2,440) = 3.627, *p* < .027, *η*^2^ = 0.027, 95% CI [0.000, 0.063]. In particular, we found that participants were more likely to accept harm (the utilitarian judgment) when moral dilemmas were preceded by erotic priming (*M* = 4.205, *SD* = 1.24) than by neutral priming (*M* = 4.095, *SD* = 1.21). The statistically significant *MD* was 0.11, 95% CI [0.004, 0.217]. Conversely, the results indicate that there was no statistically significant difference between the pleasant priming condition (*M* = 4.182, *SD* = 1.27) and the neutral priming condition (*M* = 4,095, *SD* = 1.23) (*MD* = 0.087, 95% CI [0, 0.187]), nor between the erotic priming condition and the pleasant priming condition (*MD* = 0.023, 95% CI [0, 0.128]).

Furthermore, we found a statistically significant interaction between Country and Type of Dilemma *F*(1, 220) = 8.669, *p* = .004, *η*^2^ = .038, 95% CI [0.004, 0.098]. Pairwise comparisons revealed that, when evaluating personal moral judgments, Colombian participants (*M* = 4.271, *SD* = 1.218) were more likely to accept harm than Spanish subjects (*M* = 3.809, *SD* = 1.232), *F*(1,220) = 8.309, *p* = .004, *η*^2^ = .038, 95% CI [0.004, 0.096], with a statistically significant *MD* = 0.463, 95% CI [0.146, 0.779]. There were no statistically significant differences in the case of impersonal dilemmas. On the other hand, both Colombian, *F*(1,111) = 12.815, *p =* .001, *η*^2^ = .004, 95% CI [0.000, 0.015], and Spanish participants, *F*(1,111) = 69.024 *p <* .001, *η*^2^ = .018, 95% CI [0.000, 0.047] were less willing to accept harm when judging personal rather than impersonal dilemmas. It should be noted, however, that this two-way interaction effect was qualified by the three-way interaction described below.

**Fig 1:**
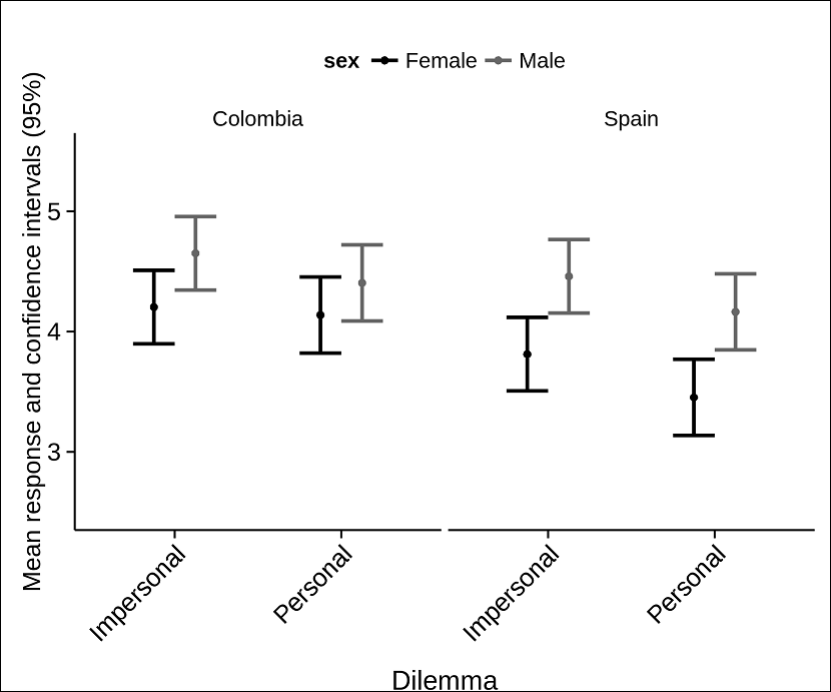
Mean responses to moral dilemmas by sex and country. Mean responses for both types of moral dilemmas (personal and impersonal) considering each sex and country (Colombia vs. Spain). Higher ratings correspond to more acceptance of harm (i.e., more utilitarian moral judgments).

Indeed, the Sex x Country x Dilemma triple interaction was statistically significant, *F*(1,220) = 4.397, *p* = .037, *η*^2^ = 0.02, 95% CI [0.000, 0.069]. Pairwise comparisons using Bonferroni-adjusted alpha levels revealed that Colombian men (*M* = 4.651, *SD* = 1.217) were more likely to accept harm than Colombian women (*M* = 4.205, *SD* = 1.139) when judging impersonal dilemmas, with an *MD* of 0.447, [0.015, 0.879], *F*(1,220) = 4.163, *p* = .043, *η*^2^ = 0.090, 95% CI [0, 0.067]. However, this was not the case for personal dilemmas, *F*(1,220) = 1.384, *p* = .241, *η*^2^ = 0.006, 90% CI [0, 0.042]. Furthermore, Colombian women were the only Country x Sex group showing no statistically significant mean differences when comparing moral judgments for personal and impersonal moral dilemmas, *F*(1,55) = 0.882, *p* = .352. By contrast, Colombian men (*F*(1,55) = 4.460, *p* < .02, *η*^2^ = .001, 95% CI [0.000, 0.021]), Spanish women (*F*(1,55) = 49.746, *p* < .001 *η*^2^ = .02, 95% CI [0.000, 0.041]), and Spanish men (*F*(1,55) = 24.013, *p* < .001, *η*^2^ = .016, 95% CI [0.007, 0.053]), preserved the double interaction described above (see Fig 1).

As in the case of Colombians, Spanish men evidenced more acceptance of harm (utilitarian judgments) than women, both for impersonal, *F* (1,220) = 8.714, *p* = .004, *η*^2^ = 0.040, 95% CI [0.004, 0.099], and personal dilemmas, *F* (1,220) = 9.811, *p =* .002, *η*^2^ = 0.045, 95% CI [0.006, 0.105]. In the former case, when comparing Spanish men (*M* = 4.459, *SD* = 1.12) and Spanish women (*M* = 3.8121, *SD* = 1.16) the MD was 0.647 (95% CI [0.215, 1.079]). When judging personal dilemmas, the mean difference between Spanish men and Spanish women was even greater (*MD* = 0.771, 95% CI [0.264, 1.158]). Note that for both types of dilemmas the effect sizes were greater than those obtained in Colombia.

Finally, when comparing men and women between countries for each type of dilemma, we found that, when judging personal dilemmas, Colombian women (*M* = 4.1378, *SD* = 1.199) were more likely to accept harm than Spanish women (*M* = 3.4532, *SD* = 1.15), *F*(1,220) = 9.097, *p* = .003, *η*^2^ = 0.04, 95% CI [0.002, 0.131], showing an *MD* of 0.685 (95% CI [0.237, 1.132]). There were no statistically significant differences between women from either of the two countries when judging impersonal dilemmas, *F*(1,220) = 3.184, *p* = .076, nor between men rating either impersonal, *F*(1,220) = 0.762, *p* = .384, or personal dilemmas, *F*(1,220) = 1.124, *p* = .29. No other factor interactions reached statistical significance at conventional alpha levels (see Table 1).

**Table 1:** Likert Means, Standard Deviations and estimated 95% Confidence Intervals for each combination of factor levels.

Country	Sex	Prime	Dilemma	Mean	Standard Deviation	95% Confidence Interval
Colombia	Female	Neutral	Impersonal	4.18	1.29	(3.85–4.52)
			Personal	4.00	1.32	(3.65–4.35)
		Pleasant	Impersonal	4.22	1.24	(3.90–4.54)
			Personal	4.14	1.31	(3.79–4.48)
		Erotic	Impersonal	4.21	1.30	(3.87–4.55)
			Personal	4.28	1.34	(3.93–4.63)
	Male	Neutral	Impersonal	4.73	1.25	(4.41–5.06)
			Personal	4.20	1.28	(3.87–4.59)
		Pleasant	Impersonal	4.59	1.36	(4.23–4.94)
			Personal	4.53	1.40	(4.16–4.89)
		Erotic	Impersonal	4.63	1.30	(4.29–4.97)
			Personal	4.49	1.40	(4.12–4.85)
Spain	Female	Neutral	Impersonal	3.72	1.28	(3.39–4.06)
			Personal	3.28	1.14	(2.98–3.58)
		Pleasant	Impersonal	3.76	1.25	(3.43–4.09)
			Personal	3.52	1.23	(3.20–3.84)
		Erotic	Impersonal	3.95	1.26	(3.62–4.28)
			Personal	3.56	1.45	(3.18–3.94)
	Male	Neutral	Impersonal	4.41	1.19	(4.10–4.72)
			Personal	4.23	1.38	(3.86–4.59)
		Pleasant	Impersonal	4.54	1.15	(4.24–4.84)
			Personal	4.16	1.40	(3.80–4.53)
		Erotic	Impersonal	4.43	1.36	(4.07–4.79)
			Personal	4.10	1.20	(3.79–4.42)

## Discussion

The main objective of the present research was to examine the effects of incidental affects, sociocultural context, type of dilemma and participant’s sex on moral judgments. On the basis of the literature reviewed, which highlighted the relevance of the above mentioned factors in moral cognition, we predicted that moral judgments would be independently influenced by each one of the considered factors. Additionally, it was predicted that the effect of suboptimal affective priming on moral judgments would vary depending on interactions with participants’ individual profiles (in terms of sex and sociocultural background) and characteristics of the target (type of dilemma).

Our results supported our main hypothesis. We found that: a) relative to neutral priming, erotic primes increased the acceptance of harm for a greater good (i.e., more utilitarian judgments); b) relative to Colombians, Spanish people rated causing harm less acceptable; c) relative to impersonal dilemmas, personal dilemmas reduced the acceptance of harmful actions; and d) relative to men, women were less likely to consider harm acceptable.

First, even though the effect of affective priming on moral judgments was not sensitive to other factors, we did find a main effect of affective priming on moral judgments. Specifically, we found that erotic (but not pleasant or neutral) primes increased the acceptance of harm. At first glance, we can interpret our results in the light of research showing that contextually induced positive affect (such as mirth) reduces preferences for deontological moral judgments [20], which is attributed to the extent that pleasant stimuli decrease negative affective reactions towards harm. On the other hand, following previous studies not related to the moral domain [43, 44], it could be inferred that the pleasant affective response to erotic primes was transferred (automatically misattributed) to moral judgments.

However, our results can hardly be explained solely in terms of a valence-based effect. For instance, prior studies [45] showing that induced moral elevation (a positive affective response) increased deontological judgments put into question the validity of a valance-based effect on moral inclinations. More importantly, the fact that the priming effect was limited to the erotic condition (but not the pleasant condition) could be due to the erotic priming having higher values in the arousal dimension. It could also be explained in the light of research on erotic priming, which suggests that effects of suboptimally presented erotic stimuli in cognition are highly specific [29, 30, 41].

With regard to the arousal hypothesis, neuroimaging data suggest that subliminal exposure to erotic stimuli increases activation in regions of the brain associated with sexual arousal [30]. Interestingly, there is evidence that sexual arousal interfered with the decision making processes under ambiguity [46] and favored a utilitarian pattern of response [33]. Consequently, it might be argued that the fact that erotic priming facilitates the acceptance of harmful actions is due to the experience of (implicitly induced) sexual arousal in the participants, which, in line with previous results [33] would facilitate a utilitarian pattern of moral judgments. Given the fact that we did not include any measure of sexual arousal, this hypothesis needs to be addressed by further research.

Indeed, it is important to notice that, when depicting erotic scenes, normative values for both valence and arousal of IAPS pictures differ significantly between men and women. In particular, erotic pictures are rated as more pleasant and more arousing in men than in women (S1 Text, see also [31–34]). However, given that we did not find that participants’ sex modulated the effect of erotic primes on moral judgments, our results suggest that the effects of erotic primes were not sensitive to sex differences in the valence and arousal values of erotic pictures. This finding might be interpreted in the light of previous research on subliminally presented erotic stimuli, which showed that the pattern of correlations between this type of exposure to erotic pictures and subjective ratings was inconsistent [28, 30]. Moreover, the fact that there is no difference between erotic and pleasant primes (which have similar arousal values to those of neutral primes), suggests that neither valence nor arousal by themselves can fully explain the obtained effect

Another possibility is that erotic primes influenced moral intuitions related to mind perception. There is evidence suggesting that erotic stimuli reduce the perception of agency (and, as a result, the moral responsibility of the agent) but also increase the perception of experience (which increases the perceived harm suffered by the victim) [47]. Based on these findings, our results would suggest that the effects of erotic primes on mind perception were focused on the dimension of agency. In particular, our results suggest that a reduction in the agent’s perceived moral responsibility would increase moral acceptability of the narrated harmful actions.

An alternative explanation comes from a process dissociation approach, which states that the strength of deontological and utilitarian inclinations within individuals can be independently measured [48]. Therefore, the fact that erotic primes increase the acceptability of harm might result from an increase or decrease in utilitarian or deontological inclinations, respectively. As mentioned above, the results of Ariely and Loewenstein [33] suggest that sexual arousal narrows motivation towards a goal state, which might increase utilitarian inclinations. Alternatively, we should consider the possibility that erotic stimuli reduced both deontological and utilitarian response tendencies; increasing the acceptability of harmful actions in incongruent moral dilemmas (which pit deontological vs. utilitarian inclinations) such as the ones used in this study [48].

Secondly, this research was designed to address the role of cultural differences in moral judgments. Our results confirmed that responses to moral dilemmas were susceptible to the “Country” factor, suggesting the presence of cultural differences in the pattern of response to moral dilemmas. In particular, we found that although there were no significant differences between countries in impersonal moral judgments, Colombian women were more likely to accept harm than Spanish women in the case of personal moral dilemmas. Indeed, Colombian women’s moral judgments were similar in the case of personal and impersonal dilemmas, evidencing different moral criteria than the Spanish sample, which did made a clear distinction between both types of moral dilemmas.

Thirdly, we found that the type of moral judgment (deontological *vs*. utilitarian) was influenced by the type of dilemma, with participants being less likely to accept harm in the case of personal dilemmas than in the case of impersonal dilemmas. This finding is congruent with previous research on the personal/impersonal distinction. As mentioned above, it is assumed that relative to impersonal dilemmas, moral judgments of personal dilemmas are characterized by a major involvement of emotional circuits, which typically leads to more deontological moral judgments [2, 49].

Finally, an important aim of the present research was to test whether sex differences interacted with additional factors such as affective priming and cultural background (country) in the making of moral judgments. We found that sex has a relevant effect on moral judgments, to the point that, across all conditions, women were less likely to accept harm than men. Our results support the dominant view in research on sex differences in moral judgments, which claims that, relative to men, women have stronger moral concerns about harm and evidence a more deontological pattern of moral judgments [4, 23]. With regard to this claim, it is important to acknowledge that, although sex differences in empathy seems to be sensitive to methodological considerations [50], several studies have found that women often perform better on tests of empathy, social sensitivity, and emotion recognition than men [51–53]. Moreover, neuroimaging studies suggest that women recruit areas containing mirror neurons to a higher degree than men, suggesting that neural circuits underlying empathy are differentially modulated by sex [54].

The present study has some limitations, and the consideration of these should help refine future research. For instance, we did not include any measure of socioeconomic status, which is known to play a role in moral judgments [6]. In addition, it is worth mentioning that, although IAPS normative values are generally consistent between Colombia and Spain, differences were identified in the dimension of arousal [39]. Nevertheless, it is important to remain cautious about normative differences of this kind, given the fact that erotic pictures validated in both Spain and Colombia are only a small set and are also partially different.

In conclusion, our results support the claim that sex, culture and incidental affect are crucial factors in moral cognition, and that the particular ways in which these factors interact shape moral judgments. On the basis of these results, further studies should explore the effects of such factors in non-moral domains, such as social judgments or aesthetic judgments. We also consider that future studies including a clinical population could improve our understanding of the role of individual differences and the ways in which they interact with contextual factors in the process of making moral judgments.

## Supporting Information

S1 Table Individual-level data (XLSX)

S1 Text S1 Appendix: Affective primes. (DOC)

S2 Text S2 Appendix: Personal and Impersonal moral dilemmas. (DOCX)
