# Supplementary material for: Effects of Suboptimally Presented Erotic Pictures on Moral Judgments: A Cross-Cultural Comparison
Source: PLoS One. 2016 Jul 1;11(7):e0158690. doi: 10.1371/journal.pone.0158690 (PMC4930184; doi:10.1371/journal.pone.0158690)
Supplement: S1 Text — (DOC) [file pone.0158690.s002.doc]

**S1 Appendix: Affective primes**

**(A) Sexual pictures from IAPS***

**Slide No:**

4647, 4607, 4311, 4651, 4652, 4656, 4658, 4659, 4660, 4664, 4668, 4670, 4672, 4677

* Selected images were characterized by their high normative values in both valence (*M*= 6.73) and arousal (*M=*6.59*).* (Lang, Ohman, & Vaitl, 1988; adapted to Spanish population by Moltó et. al., 1999 & Vila et al., 200, and to Colombian population by Gantiva, Guerra, & Vila, 2011).

Means for each picture in the IAPS for Male subjects:

4647(V**=7.25;A***=7.04),4607(V=7.99;A=7.19),4311(V=7.56;A=7.35), 4651(V=7.52; A=6.96), 4652(V=7.92; A=7.25), 4656 (V=7.21; A=6.46),4658 (V=7.35; A=6.89), 4659 (V=7.70; A=7.43),4660 (V=7.63; A=6.92), 4664 (V=7.99; A=7.72), 4668 (V=7.34; A=7.66),4670 (V=7.77; A=7.17), 4672 (V=6.44; A=6.42), 4677 (V=6.53; A=5.97)

Valence Mean = 7.44; Arousal Mean= 7.03

Means for each picture in the IAPS for Female subjects:

4647(V*=5.02;A**=5.69),4607(V=6.25;A=5.59),4311(V=5.89;A=6.08), 4651(V=5.15; A=5.71), 4652(V=5.65; A=5.98), 4656 (V=6.44; A=6.38),4658 (V=6.08; A=6.16), 4659 (V=6.15; A=6.47),4660 (V=7.22; A=6.31), 4664 (V=5.42; A=5.87), 4668 (V=6.31; A=6.85),4670 (V=6.40; A=6.42), 4672 (V=5.60; A=6.17), 4677 (V=6.63; A=6.38)

Valence Mean = 6.01; Arousal Mean= 6.15

**V: Valence Mean

***A: Arousal Mean

**(B) Pleasant pictures from IAPS** *

**Slide No**.

1440, 1463, 1710, 1721, 1920, 2209, 2655, 5831, 7220, 1460, 1620, 5600, 7220, 1731

* Selected images were characterized by their high normative values in valence (*M*= 7.71) and middle values in arousal (*M=*4.41*).* (Lang, Ohman, & Vaitl, 1988; adapted to Spanish population by Moltó et. al., 1999 & Vila et al., 200, and to Colombian population by Gantiva, Guerra, & Vila, 2011).

**(C) Neutral pictures from IAPS** *

**Slide No**.

1313, 1350, 1616, 1903, 1908, 1935, 1945, 3005.2, 5455, 5535, 5994, 7037, 7038, 7095

* Selected images were characterized by their middle normative values in both valence (*M*= 4.67) and arousal (*M=*4.3*).* (Lang, Ohman, & Vaitl, 1988; adapted to Spanish population by Moltó et. al., 1999 & Vila et al., 200, and to Colombian population by Gantiva, Guerra, & Vila, 2011).
