# Supplementary material for: Effects of Suboptimally Presented Erotic Pictures on Moral Judgments: A Cross-Cultural Comparison
Source: PLoS One. 2016 Jul 1;11(7):e0158690. doi: 10.1371/journal.pone.0158690 (PMC4930184; doi:10.1371/journal.pone.0158690)
Supplement: S2 Text — (DOCX) [file pone.0158690.s003.docx]

**S2 Appendix: Personal and Impersonal moral dilemmas**

**(Christensen, Flexas, Calabrese, Gut, & Gomila, 2014)**

Spanish version

1A) Personal

Un tranvía fuera de control se dirige a toda velocidad por las vías hacia cinco trabajadores, que morirán si el tranvía sigue su curso. Tú estás junto a las vías pero demasiado lejos para avisarlos. Junto a ti hay un desconocido muy grande.

Si empujas al desconocido a las vías, el tranvía descarrilará al atropellarle y no seguirá su curso hacia los trabajadores. Esto matará al desconocido, pero salvarás a los cinco trabajadores.

¿Haces descarrilar el tranvía empujando el desconocido a las vías, para que el tranvía no alcance a los cinco trabajadores?

1B) Impersonal

Un tranvía fuera de control se dirige a toda velocidad por las vías hacia cinco trabajadores que morirán si el tranvía sigue su curso. Tú estás junto a las vías pero demasiado lejos para avisarlos. Junto a ti hay una palanca de control que puede reconducir el tranvía a otra vía donde hay un único trabajador.

Si accionas la palanca, redirigirás el tranvía hacia la vía donde hay un trabajador, y no seguirá su curso hacia los cinco trabajadores. Esto matará a ese trabajador, pero salvará a los otros cinco.

¿Rediriges las vías accionando la palanca, cosa que hará que un trabajador sea atropellado, para que el tranvía no siga su curso hacia los otros cinco trabajadores?

2A) Personal

Soldados enemigos han ocupado tu pueblo y matarán a todos los civiles mayores de dos años. Tú y diez vecinos os refugiáis en dos habitaciones del sótano de una gran casa. Oís voces de soldados que han entrado a buscar cosas de valor. Tu bebé empieza a llorar fuertemente. Su llanto llamará la atención de los soldados, que le perdonarán la vida, pero os matarán a ti y a los otros diez refugiados.

Si le tapas la boca con la mano, amortiguarás el llanto, pero el bebé se quedará sin aire. Esto lo matará, pero os salvará a ti y a los diez vecinos.

¿Amortiguas el llanto manteniendo la mano en la boca de tu bebé, para que los soldados no os encuentren a ti y a los diez vecinos?

2B) Impersonal

Soldados enemigos han ocupado tu pueblo y matarán a todos los civiles mayores de dos años. Tú y diez vecinos os refugiáis en dos habitaciones del sótano de una gran casa. Oís voces de soldados que han entrado a buscar cosas de valor. Tu bebé empieza a llorar fuertemente. Su llanto llamará la atención de los soldados, que perdonarán la vida de tu bebé, pero os matarán a ti y a los otros diez refugiados.

Si activas una estruendosa caldera que hay se producirá un incómodo calor para los adultos, que será mortal para tu bebé, pero amortiguará su llanto, cosa que os salvará a ti y a los diez vecinos.

¿Amortiguas el llanto de tu bebé, activando la caldera que le asfixiará, para que no os encuentren a ti y a los diez vecinos?

3A) Personal

Eres parte de la tripulación de un submarino que navega debajo de un gran iceberg. Una explosión ha dañado la nave, hiriendo a varios compañeros y colapsando el único acceso entre las secciones superior e inferior. Tú y otros diez supervivientes estáis en la sección superior, que no tiene suficiente oxígeno para manteneros con vida hasta salir a la superficie. En la sección inferior, donde hay suficiente oxígeno, yace inconsciente un único compañero.

Si empujas la compuerta de emergencia entre ambas secciones abrirás el paso del aire. Sin embargo, la compuerta caerá sobre el compañero de abajo, matándolo, pero esto os salvará a ti y a los otros diez supervivientes.

¿Abres el paso del aire empujando la compuerta que caerá sobre el compañero, para que tú y los otros diez supervivientes tengáis suficiente oxígeno?

3B) Impersonal

Eres parte de la tripulación de un submarino que navega debajo de un gran iceberg. Una explosión ha dañado la nave, hiriendo a varios compañeros y colapsando el único acceso entre las partes superior e inferior. Tú y otros diez supervivientes estáis en la sección superior, que no tiene suficiente oxígeno para manteneros con vida hasta salir a la superficie. En la sección inferior, donde hay suficiente oxígeno, yace inconsciente un único compañero.

Si pulsas un interruptor de emergencia se abrirá una compuerta entre ambas secciones, dejando pasar el aire. Sin embargo, la puerta caerá sobre el compañero, matándolo, pero esto os salvará a ti y a los otros diez supervivientes

¿Abres el paso del aire pulsando el interruptor, cosa que hará caer la compuerta sobre el compañero, para que tú y los otros diez supervivientes tengáis suficiente oxígeno?

4A) Personal

Tú y diez submarinistas formáis parte de un equipo de las Naciones Unidas que desactiva minas submarinas de la IIª Guerra Mundial. Un miembro de tu equipo se ha herido y la sangre ha atraído a varios tiburones. Tienes un fusil submarino, pero sólo un arpón, y hay muchos tiburones. El submarinista que sangra está nadando hacia la última jaula protectora y la alcanzará antes que tú y los demás. Los tiburones, siguiendo la sangre, se acercan demasiado como para que tú y los otros submarinistas podáis escapar.

Si disparas al submarinista herido, esto lo matará y los tiburones se detendrán a comérselo, pero tú y los otros submarinistas podréis salvaros.

¿Haces que los tiburones se coman al submarinista herido disparándole, para que tú y los otros nueve submarinistas podáis alcanzar la jaula protectora?

4B) Impersonal

Tú y diez submarinistas formáis parte de un equipo de de las Naciones Unidas que desactiva minas submarinas de la IIª Guerra Mundial. Un miembro de tu equipo se ha herido y la sangre ha atraído a varios tiburones. Tienes un fusil submarino, pero sólo un arpón, y hay muchos tiburones. El submarinista que sangra está nadando hacia la última jaula protectora y la alcanzará antes que tú y los demás. Los tiburones, siguiendo la sangre, se acercan demasiado como para que tú y los otros submarinistas podáis escapar.

Si detonas por control remoto una de las minas, esto lo matará y los tiburones se pararán a comérselo, pero podréis salvaros tú y los otros submarinistas.

¿Haces que los tiburones se coman al submarinista herido detonando por control remoto la mina próxima a ese submarinista, para que tú y los otros nueve submarinistas podáis alcanzar la jaula protectora?

5A) Personal

Trabajas en un orfanato de un país en guerra que acoge diez niños. Los rebeldes han tomado el gobierno y están matando a todos los que no apoyan a su régimen y además llevan a cabo una campaña genocida contra un determinado grupo étnico. Tú y los diez huérfanos no apoyáis la rebelión y además uno de ellos pertenece al grupo étnico perseguido. No hay forma de que tú y los huérfanos podáis escapar y seguro que los soldados os matarán a todos.

Si asfixias con una almohada al niño del grupo étnico perseguido mientras duerme, los soldados creerán que apoyas su régimen. Esto lo matará, pero os salvará a ti a y a los otros nueve niños.

¿Haces que los soldados crean que apoyas su régimen asfixiando a dicho niño, para salvar la vida de los otros nueve niños y la tuya?

5B) Impersonal

Trabajas en un orfanato de un país en guerra que acoge diez huérfanos. Los rebeldes han tomado el gobierno y están matando a todos los que no apoyan su régimen y además llevan a cabo una campaña genocida contra un determinado grupo étnico. Tú y los diez huérfanos del orfanato no los apoyáis y además uno de los niños pertenece al grupo étnico perseguido. No hay forma de que tú y los huérfanos podáis escapar y seguro que los soldados os matarán a todos.

Si ordenas a la enfermera que le dé al niño del grupo étnico perseguido una sobredosis letal de píldoras para dormir, los soldados creerán que apoyas su régimen. Esto lo matará, pero os salvará a ti a y a los nueve niños restantes.

¿Haces que los soldados crean que apoyas su régimen ordenando a la enfermera que dé una sobredosis a dicho niño, para que os perdonen la vida a ti y a los otros nueve niños?

6A) Personal

Eres trabajador/a de la salud voluntario/a en un pueblo rural africano. Un hombre de un pueblo vecino ha contraído el virus del Ébola, que es extremadamente contagioso, incurable y casi siempre mortal. Milagrosamente, ha sobrevivido un mes, por lo que debe ser inmune a los efectos mortales del virus. Sin embargo, él cree erróneamente que tu centro de salud puede curarlo por completo. Lo ves aproximándose y sabes que si entra en el pueblo contagiará a los 119 habitantes inocentes que, a diferencia de él, morirán.

Si le disparas con la pistola del centro prevendrás que os contagie el virus a ti y al resto del pueblo. Esto lo matará, pero te salvará a ti y a los 119 habitantes del pueblo.

¿Impides que el hombre entre en el pueblo, disparándole para que no os contagie a ti y a los 119 habitantes del pueblo?

6B) Impersonal

Eres trabajador/a de la salud voluntario/a en un pueblo rural africano. Un hombre de un pueblo vecino ha contraído el virus del Ébola, que es extremadamente contagioso, incurable y casi siempre mortal. Milagrosamente, ha sobrevivido un mes, por lo que debe ser inmune a los efectos mortales del virus. Sin embargo, él cree erróneamente que tu centro de salud puede curarlo por completo. Lo ves aproximándose y sabes que si entra en el pueblo contagiará a los 119 habitantes inocentes que, a diferencia de él, morirán.

Si avisas al consejo del pueblo le dispararán, para prevenir que os contagie el virus a ti y al resto del pueblo. Esto lo matará, pero te salvará a ti y a los 119 habitantes del pueblo.

¿Impides que el hombre entre en el pueblo avisando al consejo de que le dispare, para que no os contagie a ti y a los 119 habitantes?

7A) Personal

Eres el/la único/a médico/a de un helicóptero de rescate en una inundación devastadora. Has rescatado a nueve personas heridas en estado crítico y las mantienes vivas. De pronto, el piloto te notifica que hay un fallo en el motor y que hay que reducir carga o el helicóptero se estrellará y todos moriréis. Tú no puedes abandonar el helicóptero porque los heridos necesitan tus cuidados y no puedes lanzar el pesado equipo médico porque está atornillado.

Si lanzas a uno de los heridos, liberarás carga suficiente para mantener el helicóptero en el aire. Esto lo matará, pero os salvará a ti, al piloto, y a los otros ocho heridos.

¿Reduces carga lanzando a uno de los heridos, para mantener el helicóptero en el aire, y salvar las otras diez personas a bordo?

7B) Impersonal

Eres el/la único/a médico/a de un helicóptero de rescate en una inundación devastadora. Has rescatado nueve personas heridas estado crítico y las mantienes vivas. De pronto, el piloto te notifica que hay un fallo en el motor y que hay que reducir carga o el helicóptero se estrellará y todos moriréis. Tú no puedes abandonar el helicóptero porque los heridos necesitan tus cuidados, ni lanzar el pesado equipo médico porque está atornillado.

Si cortas el cable por el que asciende la última persona rescatada, reducirás peso suficiente para mantener el helicóptero en el aire. Esto lo matará, pero os salvará a ti, al piloto y a los otros ocho heridos a bordo.

¿Liberas peso cortando el cable por el que asciende el último de los heridos, para mantener el helicóptero en el aire y salvar las otras diez personas a bordo?

8A) Personal

Tú y un compañero de investigación habéis descubierto una nueva fuente de energía que es barata, limpia y segura. Podría acabar con la contaminación y la pobreza en el mundo. Sin embargo, tu colega quiere vender el descubrimiento, y sabes que hoy planea contactar con potenciales compradores. También sabes con seguridad que éstos intentarán usar la energía como un arma horrible, pero tu compañero no te cree.

Si envenenas a tu compañero con un veneno que se encuentra en vuestro laboratorio de forma habitual, evitarás que el descubrimiento caiga en malas manos. Esta sustancia matará a tu compañero, pero salvarás a miles de personas.

¿Impides la venta del descubrimiento envenenando a tu compañero, para que la fuente de energía no se use como arma contra miles de personas?

8B) Impersonal

Tú y un compañero de investigación habéis descubierto una nueva fuente de energía que es barata, limpia y segura. Podría acabar con la contaminación y la pobreza en el mundo. Sin embargo, tu colega quiere vender el descubrimiento, y sabes que hoy planea contactar con potenciales compradores por correo electrónico. También sabes con seguridad que intentarán usar la energía como un arma horrible, pero tu compañero no te cree.

Si liberas un gas inflamable en el laboratorio, habrá una explosión en el momento en que tu compañero encienda su ordenador. Esto lo matará, pero salvarás a miles de personas.

¿Haces explotar el ordenador de tu compañero liberando el gas inflamable, para evitar que el descubrimiento se use como arma contra miles de personas?

9A) Personal

Eres inspector/a en una planta de energía nuclear. El director y tú estáis visitándola cuando uno de los conductos de abastecimiento se sobrecalienta. El sistema refrigerador de emergencia falla y está a punto de empezar una reacción en cadena que resultará en fusión nuclear. La radiación liberada será letal para una ciudad cercana, matando a miles de personas.

Si empujas al director al lugar donde confluyen los conductos de abastecimiento, su cuerpo absorberá suficiente calor para detener la reacción en cadena. Esto lo matará, pero os salvará a ti y a los miles de habitantes de la ciudad.

¿Enfrías los conductos de abastecimiento empujando al director al lugar donde confluyen, para evitar un accidente nuclear que te mataría a ti y a miles de personas?

9B) Impersonal

Eres inspector/a en una planta de energía nuclear. El director y tú estáis visitándola cuando uno de los conductos de abastecimiento se sobrecalienta. El sistema refrigerador de emergencia falla y está a punto de empezar una reacción en cadena que resultará en fusión nuclear. La radiación liberada será letal para una ciudad cercana, matando a miles de personas.

Si insertas manualmente nitrógeno líquido en la cámara de abastecimiento se eliminará suficiente calor para detener la reacción en cadena. Esto matará a un empleado atrapado en la cámara, pero os salvará a ti y a los miles de habitantes de la ciudad.

¿Eliminas calor insertando nitrógeno líquido en la cámara de abastecimiento donde se encuentra un empleado, para evitar un accidente nuclear que te mataría a ti y a miles de personas?

10A) Personal

Eres el experto/a en explosivos de una compañía encargada de demoler un rascacielos. Estás examinando la última carga cuando ves a un chico que está a punto de detonar accidentalmente una de las cargas. La explosión resultará en el derrumbe incontrolado del edificio sobre ti, el chico y la multitud de espectadores. El chico está varios pisos por debajo de ti y no puede oírte.

Si dejas caer un pesado bloque de hormigón sobre su cabeza, esto lo matará, pero os salvará a ti y a la multitud porque evitará la explosión.

¿Detienes al chico dejando caer el bloque sobre su cabeza, para que el edificio no se derrumbe sobre ti y la multitud de espectadores?

10B) Impersonal

Eres el experto/a en explosivos de una compañía encargada de demoler un rascacielos. Estás examinando la última carga cuando ves a un chico que está a punto de detonar accidentalmente una de las cargas. La explosión resultará en el derrumbe descontrolado del edificio sobre ti, el chico y la multitud de espectadores. El chico está varios pisos por debajo de ti y no puede oírte.

Si activas el diferencial devolviendo la electricidad al edificio, el chico se electrocutará dado que está tocando un circuito abierto. Esto lo matará, pero os salvará a ti y a la multitud porque evitará la explosión.

¿Detienes al chico, haciendo que se electrocute mediante la activación del diferencial, para que el edificio no se derrumbe sobre ti y la multitud de espectadores?

11A) Personal

Eres trabajador/a de la construcción. Tú y tu equipo trabajáis sobre un andamio en un rascacielos. De pronto el andamio se derrumba parcialmente. Tú y cinco compañeros estáis suspendidos de un travesaño colgante, pero pesáis demasiado para que resista. Otro compañero patina del travesaño y se agarra de tu brazo. La estructura está a punto de ceder.

Si golpeas repetidamente a ese compañero para que te suelte y caiga, esto restará suficiente peso para mantener el andamio. La caída lo matará, pero os salvará a ti y a los otros cinco compañeros.

¿Liberas peso golpeando a ese compañero, que se caerá, para que tú y los otros cinco compañeros podáis quedar colgando del travesaño?

11B) Impersonal

Eres trabajador/a de la construcción. Tú y tu equipo trabajáis sobre un andamio en un rascacielos. De pronto el andamio se derrumba parcialmente. Tú y cinco compañeros estáis suspendidos de un travesaño colgante, pero pesáis demasiado para que resista. Otro compañero patina del travesaño y se agarra de otra parte del andamio. La estructura está a punto de ceder.

Si quitas el pasador que sujeta la parte dónde está ese compañero, éste se caerá. Esto restará suficiente peso para mantener el andamio. La caída matará al compañero, pero os salvará a ti y a los otros cinco compañeros.

¿Restas el peso del compañero quitando el pasador, para que tú y los otros cinco compañeros podáis quedar colgando del travesaño?

12A) Personal

Eres conductor/a de un autocar de guardería de excursión al zoo. De camino, un defecto en las ruedas provoca que os precipitéis desde un puente a un río bravo. Tú y tres niños estáis todavía en el vehículo que se hunde. El resto han sido arrastrados por el río. Coges a los dos niños que tienes al lado y nadas hacia la salida, pero el tercer niño se agarra a tu pierna. No eres lo suficientemente fuerte para luchar contra la corriente y nadar con los tres niños.

Si te deshaces del niño que se ha agarrado a tu pierna podrás luchar contra la corriente, pero el niño se quedará atrás ahogándose. Esto lo matará, pero os salvará a ti y a los otros dos niños.

¿Luchas contra la corriente deshaciéndote del niño, cosa que hará que ese niño se ahogue, para que tú y los otros dos niños podáis alcanzar la superficie?

12B) Impersonal

Eres conductor/a de un autocar de guardería de excursión al zoo. De camino, un defecto en las ruedas provoca que os precipitéis desde un puente a un río bravo. Tú y tres niños estáis todavía en el vehículo que se hunde. El resto han sido arrastrados por el río. Coges a los dos niños que tienes al lado y nadas hacia la salida, pero está bloqueada por algunos asientos destrozados.

Si quitas los asientos liberarás la salida, pero obstaculizarás el pasillo y el tercer niño se quedará atrapado, ahogándose. Esto lo matará, pero os salvará a ti y a los otros dos niños.

¿Liberas la salida poniendo los asientos en el pasillo, cosa que atrapará al tercer niño, para que tú y los otros dos niños podáis alcanzar la superficie?

13A) Personal

Eres doctor/a. Tienes cinco pacientes que van a morir si no reciben el trasplante inmediato de algún órgano, distinto en cada caso. Otra persona llega al hospital después de un serio accidente de coche. Su estado es crítico, pero es probable que puedas salvarla mediante una larga y complicada operación.

Si cortas a propósito su arteria carótida durante la cirugía causarás su muerte sin que nadie se entere y dispondrás de los órganos para los otros cinco pacientes. Esto matará a la víctima del accidente pero salvará a los otros cinco pacientes.

¿Obtienes los órganos cortando la arteria carótida de la víctima del accidente, para que puedas realizar los trasplantes a los otros cinco pacientes?

13B) Impersonal

Eres doctor/a. Tienes cinco pacientes que van a morir si no reciben el trasplante inmediato de algún órgano, distinto en cada caso. Otra persona llega al hospital después de un serio accidente de coche. Su estado es crítico pero es probable que puedas salvarla. Durante la larga y complicada operación ves que una enfermera va a inyectarle una medicación inadecuada, letal en ese caso.

Si dejas que la enfermera inyecte la medicación errónea, la víctima del accidente morirá y dispondrás de los órganos necesarios para los otros cinco pacientes. Esto matará a la víctima, pero salvará a los cinco pacientes.

¿Obtienes los órganos dejando que la enfermera inyecte la medicación errónea a la víctima del accidente, para poder realizar los trasplantes a los otros cinco pacientes?

14A) Personal

Eres un estibador que sujeta los cables de la grúa para descargar los barcos mercantes. Tú, con otros compañeros, sujetáis estos cables a un contenedor y montáis sobre él para asegurar que se descarga adecuadamente. De pronto ves la luz roja que indica que el cable va a fallar. Te das cuenta de que la culpa la tienen dos compañeros que se están peleando. Hacen que el contenedor se balancee peligrosamente sobre otros cinco compañeros que están en el muelle.

Si empujas a los que se pelean para separarlos evitarás que el contenedor caiga, pero uno de ellos no lleva su arnés de seguridad y caerá. Esto lo matará pero salvará a los cinco compañeros de abajo.

¿Paras el balanceo del contenedor empujando a los que se pelean, cosa que hará que el compañero sin arnés caiga, para evitar que el contenedor se desplome sobre los cinco compañeros de abajo?

14B) Impersonal

Eres un estibador que sujeta los cables de la grúa para descargar los barcos mercantes. Tú, con otros compañeros, sujetáis estos cables a un contenedor y montáis sobre él para asegurar que se descarga adecuadamente. De pronto ves la luz roja que indica que el cable va a fallar. Si lo hace sobre la cubierta, el contenedor caerá sobre cinco tripulantes.

Si pulsas el botón de seguridad que soltará el contenedor de vuelta a la bodega, vosotros quedaréis suspendidos por vuestro arnés de seguridad, pero hay un miembro de la tripulación justo debajo. El desplome del contenedor lo matará, pero se salvarán a los cinco compañeros de la cubierta.

¿Pulsas el botón de seguridad para soltar el contenedor, cosa que hará que caiga sobre el tripulante de la bodega, para evitar que el contenedor caiga sobre los cinco tripulantes de la cubierta?

15A) Personal

Se ha extendido una epidemia por todo el planeta, matando a miles de personas. Eres investigador/a médico/a y has desarrollado dos sustancias en el laboratorio. Por error ambas están etiquetadas como vacuna. Una de ellas ciertamente es la vacuna, que producida en masa salvará miles de vidas. La otra es una sustancia letal.

Si inyectas esas dos sustancias, sin saber cuál es cuál, a tus dos ayudantes de laboratorio, podrás identificar la vacuna. Uno de los ayudantes morirá, pero podrás salvar miles de vidas con la vacuna.

¿Identificas la vacuna inyectando las dos sustancias a tus ayudantes, cosa que hará que uno de ellos sea envenenado, para que se puedan vacunar miles de personas?

15B) Impersonal

Se ha desatado una epidemia por todo el planeta, matando miles de personas. Eres investigador/a médico/a y has desarrollado dos sustancias en el laboratorio. Por error ambas están etiquetadas como vacuna. Una de ellas ciertamente es la vacuna, que producida en masa salvará miles de vidas. La otra es una sustancia letal.

Si permites que un enfermero inyecte esas dos sustancias, sin saber cuál es cuál, a dos pacientes, podrás identificar la vacuna. Uno de los pacientes morirá, pero podrás salvar miles de vidas con la vacuna.

¿Identificas la vacuna permitiendo que un enfermero inyecte las dos sustancias a dos pacientes, cosa que hará que uno de ellos sea envenenado, para que se puedan vacunar miles de personas?

16A) Personal

Eres un/a experta motorista participando en un evento de moteros. Mientras conduces delante de un grupo de 10 motoristas ves que otro motorista que va más adelante está perdiendo el control de su moto. Mientras aceleras para ponerte a su lado te das cuenta de que sufrirá una caída en cualquier momento, provocando un accidente en cadena en el que pueden morir los diez de los motoristas que te siguen.

Si empujas a este motorista fuera de la carretera se estrellará contra los árboles, pero evitarás el accidente en cadena. A vuestra velocidad, esto lo matará, pero salvarás a los diez motoristas.

¿Evitas el accidente en cadena empujando al motorista fuera de la carretera, salvando así la vida de los otros diez motoristas?

16B) Impersonal

Eres un/a experto/a motorista participando en un evento de moteros. Mientras conduces delante de un grupo de diez motoristas ves que otro motorista que va más adelante está perdiendo el control de su moto y cae. Puedes esquivarlo fácilmente, pero los diez motoristas detrás de ti chocarán con él y morirán en el accidente en cadena que se producirá.

Si dejas que tu moto atropelle al motorista caído, mientras saltas hábilmente a la cuneta, alertarás a tiempo a los demás. Esto matará al caído, pero salvarás a los otros diez motoristas.

¿Alertas del accidente dejando que tu moto atropelle al accidentado y saltando a la cuneta, para que los diez motoristas que te siguen también tengan tiempo de esquivarlo?

17A) Personal

Eres el líder de un pequeño grupo de soldados. Volvéis de una misión en territorio enemigo cuando uno de tus hombres cae en una trampa que atrapa su pierna, hiriéndole gravemente. No puedes liberarle sin que muera, pero si le abandonáis las tropas enemigas le torturarán hasta que revele la posición de un importante campamento aliado. Después atacarán dicho campamento y matarán al pelotón de diez hombres establecido allí. El soldado te suplica que no lo abandones, pero las tropas enemigas se acercan y es peligroso quedaros para ti y tus hombres porque habéis agotado la munición.

Si tú mismo apuñalas al soldado atrapado evitarás que sea torturado y revele la información que llevaría a la muerte de los diez soldados aliados. Esto le matará pero salvarás a los diez miembros del pelotón.

¿Impides que el soldado atrapado sea torturado apuñalándolo tú mismo para que no revele la localización de los diez integrantes del pelotón aliado?

17B) Impersonal

Eres el líder de un pequeño grupo de soldados. Volvéis de una misión en territorio enemigo cuando uno de tus hombres cae en una trampa que atrapa su pierna, hiriéndole gravemente. No puedes liberarle sin que muera, pero si le abandonáis las tropas enemigas lo torturarán hasta que revele la posición de un importante campamento aliado. Después atacarán dicho campamento y matarán al pelotón de diez soldados establecido allí. El soldado te suplica que no lo abandones, pero las tropas enemigas se acercan y es peligroso quedaros para ti y tus hombres porque habéis agotado la munición.

Si señalas la posición del soldado atrapado con una bengala, el área será bombardeada y así evitarás que el soldado sea torturado y revele la información que llevará a la muerte del pelotón aliado. Esto lo matará, pero salvarás a los diez integrantes de este pelotón.

¿Impides que el soldado atrapado sea torturado programando el bombardeo de la zona donde está, para que no revele la localización de los diez soldados aliados?

18A) Personal

Eres celador/a en un hospital durante el turno de noche. Descubres que se ha vertido un producto químico en una habitación con seis pacientes. Dicho producto es altamente tóxico y si se deja en el suelo se evapora creando gas venenoso que matará a todos los pacientes de la habitación. No se puede fregar con toallas o bayetas convencionales. Sin embargo, la piel humana lo absorbe rápidamente.

Si colocas uno de los pacientes sobre el producto su piel lo absorberá y así evitarás que se evapore. Este paciente morirá, pero salvarás a los otros cinco de la habitación.

¿Evitas la evaporación tóxica colocando a un paciente sobre el producto, para que no se intoxiquen los otros cinco pacientes?

18B) Impersonal

Eres celador/a en un hospital durante el turno de noche. Descubres que se ha vertido un producto químico en una habitación con seis pacientes. Dicho producto es altamente tóxico y si se deja en el suelo se evapora creando gas venenoso que matará a todos los pacientes de la habitación. No se puede fregar con toallas o bayetas convencionales.

Si activas el sistema de ventilación el gas venenoso será extraído de la habitación. Sin embargo, acabará en una sala del piso superior donde hay otro paciente que no puedes evacuar a tiempo. Esto matará a ese paciente, pero salvarás a los otros cinco.

¿Extraes el gas de la habitación activando el sistema de ventilación, cosa que hará que termine donde hay un paciente, para que no se intoxiquen los otros cinco pacientes?

19A) Personal

Estás en un bote de remos con un guía turístico en un lago de Alaska. Tres niños se han caído de su bote cerca de ti y ahora corren peligro de morir congelados. Rescatáis a los niños pero uno de ellos pesa mucho y si lo mantienes a bordo seguro que os hundiréis y todos moriréis. Ni tú ni el guía podéis abandonar el bote porque tú estás remando y el guía está reanimando uno de los niños.

Si tiras al niño más pesado por la borda seréis capaces de llegar a la orilla. Esto matará al niño, pero salvarás a los otros dos.

¿Eliminas peso de la barca tirando al niño más pesado, para que podáis alcanzar la orilla con los otros dos niños?

19B) Impersonal

Estás en un bote de remos con un guía turístico en un lago de Alaska. Tres niños se han caído de su bote cerca de ti y ahora corren peligro de morir congelados. . Tras rescatar dos niños te das cuenta que el peso del tercero hará que el bote se hunda y todos moriréis. Ni tú ni el guía podéis abandonar el bote porque tú estás remando y el guía está reanimando a uno de los niños.

Si vuelves a la orilla dejando al tercer niño en el agua evitarás que se hunda el bote. Esto matará al niño que dejas, pero salvarás a los otros dos.

¿Evitas el hundimiento del bote dejando atrás a un niño, cosa que hará que se congele, para que podáis alcanzar la orilla con los otros dos niños?

20A) Personal

Eres un/a electricista contratado/a para arreglar un problema en casa de un rico magnate del acero que está enfermo terminal por un cáncer. Recientemente ha sido anunciado que cuando muera donará su gran fortuna a un hospital infantil local. Sabes que hay 109 niños en ese hospital que podrían salvarse con ese dinero, pero que no pueden esperar mucho más porque están al borde de la muerte.

Si asfixias al magnate mientras duerme tapando su boca y nariz con tus manos enguantadas, avanzarás la donación vital para los niños. Esto matará al magnate, pero salvarás las vidas de 109 niños.

¿Adelantas la donación asfixiando a este hombre, para que la donación llegue a tiempo a los 109 niños enfermos?

20B) Impersonal

Eres un/a electricista contratado/a para arreglar un problema en casa de un rico magnate del acero que está enfermo terminal por un cáncer. Recientemente ha sido anunciado que cuando muera donará su gran fortuna a un hospital infantil local. Sabes que hay 112 niños en ese hospital que podrían salvarse con ese dinero, pero que no pueden esperar mucho más porque están al borde de la muerte.

Si sobrecargas los circuitos en lugar de arreglar el problema eléctrico, el soporte vital del magnate fallará y adelantarás la donación para los niños. Esto matará al magnate, pero salvarás las vidas de 112 niños.

¿Adelantas la donación haciendo que falle el soporte vital del magnate, para que la donación llegue a tiempo a los 112 niños enfermos?

21A) Personal

Eres oficial en el ejército durante una guerra. Tus soldados han encontrado un espía de alto rango atrapado y herido en un edificio parcialmente derrumbado. Te han informado por radio que los soldados enemigos pretenden un ataque sorpresa sobre una de las ciudades cercanas, matando a los miles de civiles que viven allí si tú y tus soldados no lo impiden. Seguro que el espía sabe qué ciudad será atacada pero lo has interrogado y no te lo dice. No durará mucho con vida por sus heridas.

Si lo torturas, el espía revelará la información que permitirá salvar la ciudad. La tortura lo matará, pero salvarás a miles de civiles.

¿Haces que el espía revele la información torturándole, para que puedan impedir el ataque sobre miles de civiles?

21B) Impersonal

Eres oficial en el ejército durante una guerra. Tus soldados han encontrado un espía de alto rango atrapado y herido en un edificio parcialmente derrumbado. Te han informado por radio que los soldados enemigos pretenden un ataque sorpresa sobre una de las ciudades cercanas, matando a los miles de civiles que viven allí si tú y tus soldados no lo impiden. Seguro que el espía sabe qué ciudad será atacada pero lo has interrogado y no te lo dice. No durará mucho con vida por sus heridas. Las ratas han empezado a morderlo y él agoniza.

Si permites que las ratas continúen esta tortura, el espía revelará la información que permitirá salvar la ciudad. Esto lo matará, pero salvarás a miles de civiles.

¿Haces que el espía te revele la información dejando que las ratas sigan torturándole, para que puedan impedir el ataque sobre miles de civiles?

English translations

1A) Personal

A runaway trolley is speeding down the tracks towards five workmen who will be killed if the trolley continues on its present course. You are standing next to the tracks, but you are too far away to warn them. Next to you there is a very large stranger.

If you push the large stranger onto the tracks, the trolley will slide off the tracks and won’t continue its course towards the workmen. This will kill the stranger, but you will save the five workmen.

Do you cause the trolley to derail pushing the stranger onto the tracks, so the trolley does not reach the five workmen?

1B) Impersonal

A runaway trolley is speeding down the tracks towards five workmen who will be killed if the trolley continues on its present course. You are standing next to the tracks, but you are too far away to warn them. Next to you there is a control switch that can redirect the trolley onto a different track, where only one workman is working.

If you flip the control switch it will divert the trolley onto the track with one workman and will not continue its course towards the five workmen. This will kill this workman, but it will save the other five.

Do you divert the trolley by flipping the control switch, which will cause the trolley to run over one workman, so the trolley does not reach the five workmen?

2A) Personal

Enemy soldiers have taken over your village and will kill all civilians above the age of two. You and ten neighbors are hiding in two rooms of the cellar of a large house. You can hear the voices of soldiers who have come to search the house for valuables. Your baby begins to cry loudly. The crying will attract the attention of the soldiers, who will spare your baby’s life, but will kill you and the other refugees in both rooms.

If you put your hand over its mouth the crying will be absorbed, but your baby will not be able to breathe. This will kill it, but it will save you and the other ten neighbors.

Do you absorb the crying by putting your hand over your baby’s mouth, which will leave it without air, so the soldiers won’t find you and the ten neighbors?

2B) Impersonal

Enemy soldiers have taken over your village and will kill all civilians above the age of two. You and ten neighbors are hiding in two rooms of the cellar of a large house. You can hear the voices of soldiers who have come to search the house for valuables. Your baby begins to cry loudly. The crying will attract the attention of the soldiers, who will spare your baby’s life, but will kill you and the other refugees in both rooms.

If you activate a noisy boiler it will cushion the crying, but it will become uncomfortably hot. The heat will be mortal for your baby, but it will save you and the ten neighbors.

Do you cushion the crying by activating the noisy boiler which will asphyxiate the baby, so they won’t find you and the ten neighbors?

3A) Personal

You are a crewmember on a submarine traveling under a large iceberg. An explosion has damaged the ship, injured several crewmembers and collapsed the only access between the upper and lower decks of the ship. You and ten survivors are in the upper section, which does not have enough oxygen for all of you to survive until you reach the surface. One single crewmember is lying unconscious in the lower section, where there is enough oxygen.

If you push the emergency access hatch between the upper and lower sections of the ship it will allow oxygen to flow into the upper section. However, the hatch will fall down on the crewmember in the lower section and kill him, but it will save you and the ten survivors.

Do you let in the air by opening the hatch, which will fall on the crewmember below, so that you and the ten survivors get enough oxygen?

3B) Impersonal

You are a crewmember on a submarine traveling under a large iceberg. An explosion has damaged the ship, injured several crewmembers and collapsed the only access between the upper and lower parts of the ship. You and ten survivors are in the upper section, which does not have enough oxygen for all of you to survive until you reach the surface. One single crewmember is lying unconscious in the lower section, where there is enough oxygen.

If you push an emergency button a hatch between the upper and lower sections of the ship will be released and will allow the oxygen to flow into the upper section. However, the hatch will fall on the crewmember in the lower section and kill him, but it will save you and the ten survivors.

Do you push the button, which will make the hatch fall down on the crewmember, so that you and the ten survivors get enough oxygen?

4A) Personal

You and ten divers are part of an U.N. team who is deactivating anti-ship mines from World War II. One team member has hurt himself and the blood in the water has attracted several sharks. You have an underwater rifle but only one harpoon and there are many sharks. The bleeding diver is swimming towards the last protective cage and will reach it before you and the others. The sharks, following the blood, are coming too close for you and the other divers to escape.

If you shoot at the injured diver this will kill him and the sharks will stop to eat him, but you and the ten divers will be saved.

Do you let the sharks eat the injured diver by shooting at him, so you and the other ten divers can reach the protective cage?

4B) Impersonal

You and ten divers are part of an U.N. team who is deactivating anti-ship mines from World War II. One team member has hurt himself and the blood in the water has attracted several sharks. You have an underwater rifle, but only one harpoon and there are many sharks. The bleeding diver is swimming towards the last protective cage and will reach it before you and the others. The sharks, following the blood, are coming too close for you and the other divers to escape.

If you detonate one of the mines by mean of a remote control, it will kill the injured diver and the sharks will stop to eat him. Then, you and the ten divers will be saved.

Do you let the sharks eat the injured diver by detonating the mine next to him with a remote control, so you and the other ten divers can reach the protective cage?

5A) Personal

You work in an orphanage in a war swept country that hosts ten orphans. Rebel soldiers have taken over the government and are now killing everyone who does not support their regime. Moreover, they are conducting an ‘ethnic cleansing’ genocide campaign to kill everyone of a particular ethnic group. You and the eleven children of the orphanage did not support the rebellion and you also have one child of this ethnic group in your orphanage. There is no way for you and the other children to escape and the soldiers will certainly kill you all.

If you smother the child of the pursued ethnic group to death with a pillow while he is sleeping, the soldiers will believe that you support their regime. This will kill the child, but it will save you and the remaining ten children.

Do you make the soldiers believe you are supporting their regime by smothering that one child, so they will spare your and the other ten children’s life?

5B) Impersonal

You work in an orphanage in a war swept country that hosts ten orphans. Rebel soldiers have taken over the government and are now killing everyone who does not support their regime. Moreover, they are conducting an ‘ethnic cleansing’ genocide campaign to kill everyone of a particular ethnic group. You and the eleven children of the orphanage did not support the rebellion and you also have one child of this ethnic group in your orphanage. There is no way for you and the other children to escape and the soldiers will certainly kill you all.

If you order a nurse to give the child of the pursued ethnic group a lethal overdose of sleeping pills, the soldiers will believe that you support their regime. This will kill the child, but it will save you and the remaining ten children.

Do you make the soldiers believe you are supporting their regime ordering the nurse to give that one child an overdose, so they will spare your and the other ten children’s life?

6A) Personal

You are a Peace Corps health-worker who is volunteering in a rural African village. A man from a nearby village has been infected with the Ebola virus that is extremely contagious, incurable, and almost always deadly. Unexpectedly, this man is still alive which means he must be immune to the virus’ deadly consequences. However, he wrongly believes that your health center can cure his disease completely. You see him approaching and you know that if he enters the village he will pass the virus to 119 innocent people who, unlike him, will die.

If you shoot him with the gun of the health center you will prevent him from spreading the virus to you and the rest of the village. This will kill him, but it will save you and the 119 inhabitants of the village.

Do you prevent the man from entering the village by shooting him, so he won’t spread the virus to you and the 119 people of the village?

6B) Impersonal

You are a Peace Corps health-worker who is volunteering in a rural African village. A man from a nearby village has been infected with the Ebola virus that is extremely contagious, incurable, and almost always deadly. Unexpectedly, this man is still alive, which means he must be immune to the virus’ deadly consequences. However, he wrongly believes that your health center can cure his disease completely. You see him approaching and you know that if he enters the village he will pass the virus to 119 innocent people who, unlike him, will die.

If you warn the village council they will shoot him to prevent him from spreading the virus to you and the rest of the village. This will kill him, but it will save you and the 119 inhabitants of the village.

Do you prevent the man from entering into the village by warning the village council so that they shoot him, so he won’t spread the virus to you and the 119 people of the village?

7A) Personal

You are a doctor alone in a rescue helicopter during a devastating flood. You have rescued eight critically injured people and you are keeping them alive. Suddenly, the pilot notifies you of a problem in the engine and gives the order that the helicopter needs to release weight or it will crash and you will all die. You cannot abandon the helicopter because the wounded need your help and the heavy medical equipment is bolted down.

If you throw off one of the injured people, you will release enough weight to keep the helicopter in the air. This will kill him, but it will save you and the eight people on board.

Do you release weight by throwing one of the injured people off the helicopter, so you and the eight persons on board will be able to stay in the air?

7B) Impersonal

You are a doctor alone in a rescue helicopter during a devastating flood. You have rescued eight critically injured people and you are keeping them alive. Suddenly, the pilot notifies you of a problem in the engine and gives the order that the helicopter needs to release weight or it will crash and you will all die. You cannot abandon the helicopter because the wounded need your help and the heavy medical equipment is bolted down.

If you cut the cable by which the last rescued person is ascending, you will release enough weight to keep the helicopter in the air. This will kill him, but it will save you and the eight people on board.

Do you release weight by cutting the cable by which the last person is ascending, in order to keep you and the eight people on board in the air?

8A) Personal

You and a fellow researcher have discovered a powerful new energy source that is cheap, safe, and clean. It has the potential to put an end to pollution and poverty in the world However, your colleague wants to sell the discovery and you know that he is planning to contact the potential buyers today. You also know for sure that the latter will use the invention as a deadly weapon, but your colleague doesn’t believe you.

If you poison your colleague with a common poison available in your lab, you will avoid having the discovery fall into the wrong hands. The poison will kill your colleague, but you will save thousands of people.

Do you prevent the sale of the discovery by poisoning your colleague, so the source of energy can’t be used as a weapon against thousands of people?

8B) Impersonal

You and a fellow researcher have discovered a powerful new energy source that is cheap, safe, and clean. It has the potential to put an end to pollution and poverty in the world However, your colleague wants to sell the discovery and you know that he is planning to contact the potential buyers by email today. You also know for sure that the latter will use the invention as a deadly weapon, but your colleague doesn’t believe you.

If you release a flammable gas in the lab, it will cause an explosion when your colleague turns on his computer. This will kill him, but you will save thousands of people.

Do you cause the computer of your colleague to explode releasing the flammable gas, in order to prevent the discovery being used as a weapon against thousands of people?

9A) Personal

You are an inspector of a nuclear power plant. The plant foreman and you are visiting the facility when one of the supply conduits overheats. The emergency coolant system fails to activate, and a chain reaction which will result in a nuclear meltdown is about to start. This will release lethal radiation into the nearby town, killing thousands of people.

If you push the foreman onto the conduits it will release enough heat to prevent the nuclear chain reaction. This will kill him, but it will save you and the thousands of town inhabitants.

Do you cool down the conduits by pushing the foreman down on them, so the nuclear chain reaction will be stopped in time for you and the thousands of people?

9B) Impersonal

You are an inspector of a nuclear power plant. The plant foreman and you are visiting the facility when one of the supply conduits overheats. The emergency coolant system fails to activate, and a chain reaction which will result in a nuclear meltdown is about to start. This will release lethal radiation into the nearby town, killing thousands of people.

If you insert liquid nitrogen manually into the supply chamber it will release enough heat to prevent the nuclear chain reaction. This will kill one employee, who is trapped in the supply chamber, but it will save you and the thousands of town inhabitants.

Do you release the heat inserting liquid nitrogen into the supply chamber where the employee is trapped, so the nuclear chain reaction will be stopped in time for you and the thousands of people?

10A) Personal

You are the explosives expert of a company that has been hired to demolish a skyscraper. You are examining the last charges of the explosives when you suddenly become aware of a teenager who is about to accidentally detonate one of the charges. The explosion will result in the building’s uncontrolled collapse over you, the teenager, and the crowd of spectators. The teenager stands several floors below you and cannot hear you.

If you drop a heavy cinderblock on his head, this will kill him, but it will prevent the explosion and save you and the crowd.

Do you stop the teenager by dropping the cinderblock on his head, so the building won’t collapse over you and the crowd of spectators?

10B) Impersonal

You are the explosives expert of a company that has been hired to demolish a skyscraper. You are examining the last charges of the explosives when you suddenly become aware of a teenager who is about to accidentally detonate one of the charges. The explosion will result in the building’s uncontrolled collapse over you, the teenager, and the crowd of spectators. The teenager stands several floors below you and cannot hear you.

If you reactivate the building’s electricity, the boy will get an electric shock because he is touching an open circuit. This will kill him, but it will save you and the crowd by preventing the explosion.

Do you stop the teenager by reactivating the building’s electricity, so the building won’t collapse over you and the crowd of spectators?

11A) Personal

You are a construction worker. You and your crew are standing on some scaffolding, working on a skyscraper. Suddenly, the scaffolding collapses partially. You and several others are hanging on to a dangling crossbar, but it cannot hold the weight of all of you. A worker next to you slips off the crossbar and grabs your one free arm. However, you realize that the entire structure is about to give way.

If you repeatedly kick your coworker in the stomach and chest until he lets go of your hand and falls, you will remove just enough weight so the rest of you can make it to safety before the scaffolding collapses entirely. This will kill your colleague but it will safe you and the others.

Do you remove weight by hitting this colleague until he lets go of your arm and falls, so you and the others can continue to hold on to the structure?

11B) Impersonal

You are a construction worker. You and your crew are standing on some scaffolding, working on a skyscraper. Suddenly, the scaffolding collapses partially. You and several others are hanging on to a dangling crossbar, but it cannot hold the weight of all of you. The worker next to you slips off the crossbar and catches himself on another portion of the scaffolding. The entire structure is about to give way.

If you pull out a latch that will detach the section of scaffolding with your co-worker on it, this will remove just enough weight that the rest of you can make it to safety before the scaffolding collapses entirely. This will kill him but save you and the other five coworkers.

Do you remove weight by pulling out the latch so your co-worker falls off the crossbar, so you and the others can continue to hold on to the structure?

12A) Personal

You are the bus driver of a kindergarten trip to the zoo. On the way, faulty tires cause the bus to plunge off a bridge and into a roaring river. You and three of the children are still in the sinking bus. The rest has already been swept away by the river. You grab the two children next to you and begin to swim towards the exit. The third child latches onto your leg. You feel that you are not strong enough to fight against the current and swim with all three children.

If you shake off the third child you will be able to fight against the current, but the child dropped behind will drown. This will kill him, but it will save you and the other two children.

Do you fight against the current shaking off the child, which will make him to drown, so you and the other two children can reach the surface?

12B) Impersonal

You are the bus driver of a kindergarten trip to the zoo. On the way, faulty tires cause the bus to plunge off a bridge and into a roaring river. You and three of the children are still in the sinking bus. The rest has already been swept away by the river. You grab the two children next to you and begin to swim towards the exit, but bulky seats are blocking it.

If you pull the seats away you will free the exit, but you will block the corridor and the third child will be trapped, causing him to drown. This will kill him, but it will save you and the other two children.

Do you free the exit putting the seats in the corridor, which will trap the third child, so you and the other two children can reach the surface?

13A) Personal

You are a doctor. You have five patients who will all die if they don’t get an immediate organ transplant. Another patient is just being rushed to the hospital after a serious car accident. This person is critically injured, but you can probably save him by means of a long and complicated operation.

If you secretly cut his carotid artery during surgery it will cause his death and you will have the organs for the other five patients. This will kill him, but save the other five patients.

Do you obtain the organs cutting the carotid artery of the accident victim, so you can undertake the transplantations for the other five patients?

13B) Impersonal

You are a doctor. You have five patients who will all die if they don’t get an immediate organ transplant. Another patient is just being rushed to the hospital after a serious car accident. This person is critically injured. During the long and complicated surgery you notice that a nurse is about to inject the wrong medication, which will be lethal in this case.

If you let the nurse inject the wrong medication the accident victim will die and you will have the organs for the other five patients. This will kill this patient, but will save the other five patients.

Do you obtain the organs letting the nurse inject the wrong medication, so you can undertake the transplantations for the other five patients?

14A) Personal

You are part of a shipyard dock team that attaches crane cables to containers to unload the cargo ships. You and the others have just attached cables to a container and are now climbing on top of it to make sure it is unloaded properly. Suddenly the red warning light flashes indicating that the crane cable is about to fail. You see that the reason for this is a fight between two crewmembers. The container is swinging dangerously over five other crewmembers on the deck.

If you push the two fighting crewmembers to separate them, you will avoid having the container fall down. However, one of the crewmembers is not wearing his safety harness and will fall off the container. This will kill him, but it will save the five crewmembers on the deck.

Do you stop the swinging of the container by pushing your colleagues, which will cause the one without safety harness to fall down, so the container won’t collapse onto the five crewmembers on the deck?

14B) Impersonal

You are part of a shipyard dock team that attaches crane cables to containers to unload the cargo ships. You and the others have just attached cables to a container and are now climbing on top of it to make sure it is unloaded properly. Suddenly the red warning light flashes indicating that a cable is about to fail. If it fails over the deck the container will collapse onto five crewmembers.

If you push the emergency release button the container will be dropped back into the cargo bay. You and the others will be held suspended in mid air by your safety harnesses, but one crewmember is still working in the cargo bay. Dropping the container back there will kill him, but it will save the five crewmembers on the deck.

Do you drop the container pushing the emergency button, which will cause the container to fall back into the cargo bay on the crewmember, so the container won’t collapse onto the five crewmembers on the deck?

15A) Personal

A viral epidemic has spread across the globe killing thousands of people. You are a medical researcher and have developed two substances in your laboratory. Due to a mistake both are labeled as vaccine. One of them is indeed the vaccine, which could save thousands of lives. The other one is lethal.

If you inject the substances to your two lab assistants without knowing which substance is which, you will be able to identify the vaccine. One of the assistants will die, but you will be able to save thousands of lives with your vaccine.

Do you identify the vaccine injecting these two substances into your two lab assistants which will poison one of them, so thousands of people can be vaccinated?

15B) Impersonal

A viral epidemic has spread across the globe killing thousands of people. You are a medical researcher and have developed two substances in your laboratory. Due to a mistake both are labeled as vaccine. One of them is indeed the vaccine, which could save thousands of lives. The other one is lethal.

If you allow a nurse to inject the two substances to two patients, you will be able to identify the vaccine. One of the patients will die, but you will be able to save thousands of lives with the vaccine.

Do you identify the vaccine allowing the nurse to inject the two substances to two patients which will poison one of them, so thousands of people can be vaccinated?

16A) Personal

You are an expert motorcyclist participating in a Bike Week. As you are driving down the road in front of a large group of ten bikers, you suddenly notice that a biker up front is losing control over his machine. As you speed up to pull alongside him, you realize that he is going to crash any moment. This would result in a large pile-up and the group of ten bikers behind you will die.

If you force this biker off the road he will crash into the trees, but you will prevent the pile-up. At your current speed this will kill him, but you will save the group of ten riders.

Do you prevent the large pile-up by forcing this biker off the road, in order to impede that the group of ten bikers crash into him?

16B) Impersonal

You are an expert motorcyclist participating in a Bike Week. As you are driving down the road in front of a large group of ten bikers, you suddenly notice that a biker up front is losing control over his machine and is falling. You can easily evade him yourself but the bikers behind you will crash into each other and die in the resulting pile-up.

If you let your bike run into the falling biker while jumping into the roadside ditch yourself, you will warn the others in time. This will kill this biker, but you will save the other ten.

Do you alert the others about the imminent accident by running your bike into the falling bikers’ machine, so the other ten riders will have enough time to drive around him?

17A) Personal

You are the leader of a small group of soldiers. You are on your way back from a mission in enemy territory when one of your men steps in a trap that catches his leg, injuring him badly. You cannot free him without killing him, but if you leave him behind, enemy troops will torture him until he reveals the position of an important allied base camp. After that the soldiers will attack this camp and kill the 10 soldiers of the platoon there. The soldier begs you not to leave him behind, but the enemy troops are approaching and it is dangerous for you and your men to stay because you don’t have any more ammunition.

If you stab the soldier yourself you will impede that he’ll be tortured and reveal the information which leads to the death of the allied platoon. This will kill him, but you will save the ten soldiers of the platoon.

Do you impede that the trapped soldier will be tortured by stabbing him to death, so he can’t reveal the location of the ten allied troop members?

17B) Impersonal

You are the leader of a small group of soldiers. You are on your way back from a mission in enemy territory when one of your men steps in a trap that catches his leg, injuring him badly. You cannot free him without killing him, but if you leave him behind, enemy troops will torture him until he reveals the position of an important allied base camp. After that the soldiers will attack this camp and kill the 10 soldiers of the platoon there. The soldier begs you not to leave him behind, but the enemy troops are approaching and it is dangerous for you and your men to stay because you don’t have more ammunition.

If you signalize the position of the trapped soldier with an illumination rocket, the region will be bombed. Thus, you will avoid that the soldier is tortured and reveals the information which will lead to the death of the allied troop. This will kill him, but you will save the ten soldiers of the allied platoon.

Do you impede that the trapped soldier is tortured by ordering the bombing of that region, so he can’t reveal the location of the ten allied troop members?

18A) Personal

You are on the night watch in a hospital. A chemical product has been spilled in a room with six patients. This chemical is highly toxic because once exposed to oxygen it evaporates and transforms into a poisonous gas which will kill all the patients in the room. You cannot wipe it away with conventional towels or wiping clothes. However, human skin absorbs it swiftly.

If you place one of the patients on the chemical you will prevent it from evaporating. This will kill this patient but you will save the other five patients in the room.

Do you prevent the toxic evaporation placing one of the patients on the chemical, so the other five patients won’t be poisoned?

18B) Impersonal

You are on the night watch in a hospital. A chemical product has been spilled in a room with five patients. This chemical is highly toxic because once exposed to oxygen it evaporates and transforms into a poisonous gas which will kill all the patients in the room. You cannot wipe it away with conventional towels or wiping clothes.

If you activate the ventilation system the poisonous gas will be taken out of the room. However, it will be transferred into a room upstairs with one patient, whom you won’t be able to evacuate in time. This will kill this patient, but you will save the other five.

Do you divert the gas out of the room by activating the ventilation system, causing it to be transferred to the room with one patient, so the other five patients won’t be poisoned?

19A) Personal

You are on a rowboat with a tour guide on a lake in Alaska. Nearby, three children have fallen off their boat and are in danger of freezing to death. You rescue the children, but one of them is very heavy and you realize that if you keep him on board your boat will sink and all will die. Neither you nor the guide can leave the boat because you are rowing and the guide is giving CPR to one of the children.

If you throw the heavy child off the boat you will be able to reach the shore. This will kill this child, but you will save the other two.

Do you get rid of load by throwing that one child overboard, so you can reach the shore with the other two children?

19B) Impersonal

You are on a rowboat with a tour guide on a lake in Alaska. Nearby, three children have fallen off their boat and are in danger of freezing to death. After you rescue two of the children, you realize that the weight of the third child would cause your boat to sink and all would die. Neither you nor the guide can leave the boat because you are rowing and the guide is giving CPR to one of the children.

If you row to the shore leaving the third child behind in the water, you will prevent your boat from sinking. This will kill the child left behind, but you will save the other two.

Do you prevent your boot from sinking by leaving the third child behind, which will cause him to die, so that you can reach the shore with the other two children?

20A) Personal

You are an electrician hired to fix an electrical problem in the home of a very wealthy tycoon who is terminally ill of cancer. Recently, it has been announced that upon his death, his enormous fortune will be donated to the local children’s hospital. You know that there are 109 children at this hospital who could be saved with this money, but who cannot wait much longer before they die.

If you suffocate the tycoon while he is sleeping by covering his nose and mouth with your gloved hands, this essential donation for the children will arrive in time. This will kill the tycoon, but you will save the lives of 109 children.

Do you speed the donation by suffocating this man, so that the donation will get to the 109 ill children in time?

20B) Impersonal

You are an electrician hired to fix an electrical problem in the home of a very wealthy tycoon who is terminally ill of cancer. Recently, it has been announced that upon his death, his enormous fortune will be donated to the local children’s hospital. You know that there are 112 children at this hospital who could be saved with this money, but who cannot wait much longer before they die.

If you short-circuit the cables instead of fixing the problem, the tycoon’s life support equipment will fail and this essential donation for the children will arrive in time. This will kill the tycoon, but you will save the lives of 112 children.

Do you anticipate the donation by causing the tycoon’s life support equipment to fail, so that the donation gets to the 112 ill children in time?

21A) Personal

You are a military officer during a war. Your soldiers have found an important enemy spy trapped and injured in a collapsed building. Over the radio you have been informed that enemy soldiers are planning a surprise attack on a nearby town, but you don’t know which. They will kill thousands of civilians if you and your soldiers do not stop them. The spy knows which town will be attacked. You have questioned him but he doesn’t want to tell you. He won’t survive much longer due to his injuries.

If you torture the spy, he will reveal the information about the town. The torture will kill him, but you will save thousands of civilians.

Do you make the spy reveal the information by torturing him, so you can prevent the attack on thousands of civilians?

21B) Impersonal

You are a military officer during a war. Your soldiers have found an important enemy spy trapped and injured in a collapsed building. Over the radio you have been informed that enemy soldiers are planning a surprise attack on a nearby town, but you don’t know which. They will kill thousands of civilians if you and your soldiers do not stop them. The spy knows which town will be attacked. You have questioned him but he doesn’t want to tell you. He won’t survive much longer due to his injuries. Rats have begun to chew his legs and he is suffering a lot.

If you let the rats continue with this torture the spy will reveal the information about the town. The torture will kill him, but you will save thousands of civilians.

Do you make the spy reveal the information by letting the rats continue to torture him, so that you can prevent the attack on the thousands of civilians?
